# 

**Published:** 1991-09

**Authors:** 


South West Vascular Surgeons

### WEST OF ENGLAND MEDICAL JOURNAL

**SEPTEMBER 1991** **Volume 106(iii)**

Copyright © West of England Medical Journal Ltd 1991.

**Contents**

58 **The Waldegrave Interview**

60 **The Bristol Royal Infirmary as a Trust Hospital — the first six months.**
Interview with Dr John Roylance

61 **Private Practice — Does it have a place?**
Ian S. Bailey

63 **Thoughts on the NHS**
M. D. Read

65 **Recreational Drowning Deaths in the South West of England**
Andrew Rouse

67 **Job-sharing in General Practice — a thing of beauty**
William Hamilton

68 **Effect of the Cornwall Helicopter Ambulance on Ambulance Service Emergency Response Time**
Andrew Rouse

72 **Malignant Neuro-endocrine tumour of the Pancreas, Salmonella Enteritidis Cholangitis and Pseudo-membranous Cholecystitis**
Andrew Skryme Jones, Stephen Hughes, Patricia Burton, Graham Appleton, David Leaper

74 **Ultrasound of the Infant Hip**
Mark Cobby, Nicholas Clarke, Andrew Duncan

77 **Why I like chest physicians**
John L. Burton

79 **From Our Correspondents**

81 **Book Reviews**
Cario-vascular System — Hartnell.
Essentials of Uro-radiology — Amis and Newhouse.
Evolution and a Creator — Beale.
The Best of Medical Humour — Bennett

83 **South Western Obstetrical and Gynaecological Society**
Meeting in Bristol May 1991

85 **South West Vascular Surgeons**
Meeting in Gloucester February 1991.

***What I know remains unknown unless by me to others shown.***
**Lucilius, (180—102 BC)**

